# Magnetic fingerprints of rolling cells for quantitative flow cytometry in whole blood

**DOI:** 10.1038/srep32838

**Published:** 2016-09-06

**Authors:** Mathias Reisbeck, Michael Johannes Helou, Lukas Richter, Barbara Kappes, Oliver Friedrich, Oliver Hayden

**Affiliations:** 1 In-Vitro DX & Bioscience, Department of Strategy and Innovation, Siemens Healthcare GmbH, Günther-Scharowsky-Str. 1, 91058 Erlangen, Germany; 2 Institute of Medical Biotechnology, Department of Chemical and Biological Engineering, Friedrich-Alexander-University Erlangen-Nuremberg (FAU), Paul-Gordan-Str. 3, 91052 Erlangen, Germany

## Abstract

Over the past 50 years, flow cytometry has had a profound impact on preclinical and clinical applications requiring single cell function information for counting, sub-typing and quantification of epitope expression. At the same time, the workflow complexity and high costs of such optical systems still limit flow cytometry applications to specialized laboratories. Here, we present a quantitative magnetic flow cytometer that incorporates *in situ* magnetophoretic cell focusing for highly accurate and reproducible rolling of the cellular targets over giant magnetoresistance sensing elements. Time-of-flight analysis is used to unveil quantitative single cell information contained in its magnetic fingerprint. Furthermore, we used erythrocytes as a biological model to validate our methodology with respect to precise analysis of the hydrodynamic cell diameter, quantification of binding capacity of immunomagnetic labels, and discrimination of cell morphology. The extracted time-of-flight information should enable point-of-care quantitative flow cytometry in whole blood for clinical applications, such as immunology and primary hemostasis.

Single cell function analysis is a hallmark for biotechnology and *in-vitro* diagnostics. Today, fluorescence flow cytometry is the dominating methodology to serve the industry and hospitals with mainly qualitative but increasingly multiplexed analysis of single cells^1^. Most recently, mass spectrometry was introduced for cell function analysis^2^. Briefly, the advantages of mass spectrometry include greater multiplexing capability and a lower background signal. However, sample preparation remains laborious and time-consuming for both methods, presenting a major drawback for an integrated workflow. In detail these systems require pre-analytical steps such as hemolysis of erythrocytes to minimize background effects, which however, can result in a significant loss of the targeted biomarkers^3^,^4^. Furthermore, the complexity of the instruments limits miniaturization. Thus, flow cytometry is limited to specialized laboratories and cannot be used for bedside testing.

However, in recent years, the integration of giant magnetoresistance (GMR) and Hall sensors in microfluidic devices for immunomagnetic detection of biological targets has become an emerging field allowing for non-optical probing of biological samples^5^,^6^,^7^,^8^,^9^,^10^,^11^,^12^,^13^,^14^,^15^. With latest advances in magnetic nanoparticle (MNP) fabrication and functionalization, a number of highly specific sub-micron sized MNPs has become commercially available leading to an increasing interest and research in the field of immunomagnetic cell separation and sensing techniques^16^. Several reports demonstrate the use of a magnetic read-out for ELISA-type assays^5^,^6^,^7^,^8^,^9^,^10^,^11^, but few reports cover single cell analysis^12^,^13^,^14^,^15^. Contrary to optical flow cytometry, magnetic detection assays can be even operated without any pre-analytical sample preparation even in whole blood as the ensemble of MNPs bound to the surface of a target cell shows a significantly higher magnetic moment than the biological matrix^17^. However, previous approaches towards magnetic flow cytometry only obtained qualitative information.

Here, we report a quantitative flow cytometry method to probe the magnetic fingerprint of the nanoscale coverage of immunomagnetically labeled cells in a solely magnetic-based and negligible background assay without the need for hemolysis of opaque whole blood. Furthermore, we show that with magnetic time-of-flight (TOF) analysis of target cells rolling over the sensor, accurate cell diameters, cell morphology, and immunomagnetic label density can be derived all at the same time in the presence of a complex background such as whole blood, which allows a completely new approach towards quantitative single cell function analysis.

## Results

### Composition and numerical model of our magnetic flow cytometer

**Figure 1:**
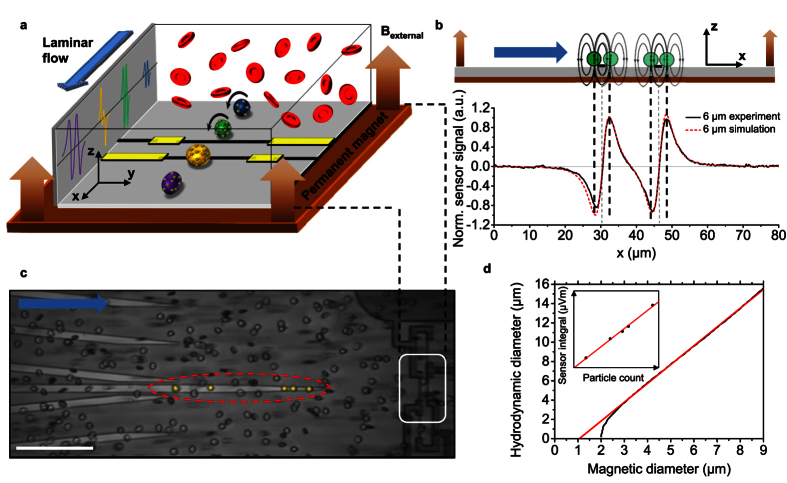
Quantitative cell detection in opaque media with integrated real-time cell function testing. (**a**) Magnetic fingerprints of immunomagnetically labeled single cells under laminar flow conditions are acquired by a Wheatstone half-bridge layout with two GMR resistors (black lines) in diagonal configuration enabling cell discrimination based on antibody binding capacity (blue), cell volume (green and yellow) and TOF (purple). (**b**) Cell positions are allocated to simulated and measured characteristic signal propagation for a homogeneously magnetized sphere of 6 μm diameter. The distance between two peaks originating from one resistor (black dashed lines) is defined as the magnetic diameter of the analyte. (**c**) *In situ* magnetophoretic cell enrichment and focusing on NiFe patterns allows for highest lateral reproducibility of the magnetically labeled target cells crossing the sensing elements (white box). Optimally focused cells are highlighted in yellow and surrounded by an oval. Scale bar is 100 μm. (**d**) Linear dependence of the magnetic diameter on the hydrodynamic diameter allows for effortless calculation of the cell volume from the magnetic fingerprint. The inset shows the linear increase in the sensor output depending on the amount of magnetic nanoparticles bound to a cell.

The schematic illustration of the magnetic flow cytometer in Fig. 1a depicts the non-optical detection scheme that allows us to perform diminished-background and quantitative analysis of immunomagnetically labeled target cells in opaque media, such as whole blood, even in the presence of excess label or red blood cells. The sensor comprises a Wheatstone bridge configuration of four 2 × 30 μm^2^ GMR spin-valve resistors encapsulated by a pin-hole free 70 nm SiN passivation layer which prevents the sensor from corrosion in ionic media and provides minimal spacing between sensor and analyte for an optimal signal-to-noise ratio. For the detection of the labeled cellular targets in a laminar flow regime, the sensors are incorporated into a microfluidic channel with a cross section of a 700 × 150 μm^2^ and a length of 15 mm. The assembled device is positioned over the center area of a 32 × 27 × 5 mm^3^ NdFeB permanent magnet generating a vertical magnetic field density B_external_ between 100 mT and 170 mT which covers the complete 20 × 10 mm^2^ Si chip area. To ensure highest sensitivity of the spin valve in the μT regime the Wheatstone half-bridge has to be precisely positioned relative to the center area of the NdFeB permanent magnet (Supplementary Fig. 1)^18^. Schematically drawn signals from a half-bridge are shown for rolling cells with high and low labeling load, different diameters, and morphology in Fig. 1a.

Key of our methodology towards quantitative single cell analysis is the TOF approach based on the characteristic signal pattern for each single cell. The signal of a single cell is recorded with a Wheatstone half-bridge oriented transversely to the laminar flow direction. The magnetic field ***B***_*sum*_ originating from an immunomagnetically labeled cell can be derived from numerical simulation by summing up the average in-plane (x-direction) component of the stray field of each MNP over the resistor area *A*_*Sensor*_^13^


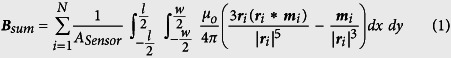


with ***m***_*i*_ as the individual magnetic moment of a nanoparticle, *N* as the number of bound MNPs per cell, *μ*_*0*_ as the magnetic field constant, *l* as the sensor length, *w* as the sensor width, and the position vector


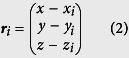


The magnetic stray field formed around a homogenously labeled sphere can be approximated by a magnetic dipole field ***B***_*dipole*_ located at the center of the analyte^19^,


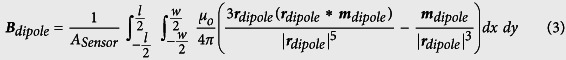


where ***m***_*dipole*_ is the sum of the magnetic moment of the bound MNPs and ***r***_*dipole*_ is the position vector. When the cell is approaching the first GMR resistor of the half-bridge, the in-plane component of the dipole field of the analyte is detected. As the detected field vectors of the immunomagnetically labeled cell are orientated in negative x-direction the sensing layer is moved towards an antiparallel state to the hard layer that is pinned in positive x-direction. Hence, the signal decreases, resulting in the first minimum of the sensor signal. As the cell travels further over the sensor the signal begins to increase until it returns to its original base line value in the magnetic zero field. This position can be assigned to the cell located directly above the resistor as the positive and negative parts of the dipole field of a homogenously labeled sphere eliminate each other. As the cell is passing the sensor element the soft magnetic layer of the sensor system is moved into the opposite direction due to the inverted in-plane component being detected. This causes a maximum in the differential signal of the Wheatstone bridge. For a homogenous magnetization the two signal half-waves can be considered symmetric around the base line crossing between the peaks. The signal is duplicated with the half-bridge configuration, resulting in the characteristic four-peak-pattern (Fig. 1b). Our TOF approach utilizes the specific positions of the cell relative to the GMR stripes in order to transfer the signal pattern into the distance domain. With the known distance of the resistors of the half-bridge and the measured rolling time, the mean velocity for each cell is derived. The normalized signal derived from numerical simulation is in good agreement with a 6 μm analyte, magnetized on its surface, passing a sensor with a 16 μm distance between the two GMR resistors at a velocity of 800 μm s^−1^ (Fig. 1b).

### *In situ* magnetophoretic focusing for precise signal analysis

Controlled focusing of the analytes rolling on the substrate surface over the center sensing elements constitutes an essential part of the presented magnetic TOF measurement (Supplementary Movie 1). First, we enrich the stochastically distributed cells in z-direction. The NdFeB permanent magnet placed underneath the sensor pulls the labeled cells onto the substrate surface, where they are laterally enriched to the center of the microfluidic channel by ferromagnetic NiFe fishbone-like chevron patterns located on the substrate surface^20^. These chevrons with a thickness between 70 nm and 350 nm, a width of 5 μm for a diameter range of 3 μm to 8 μm cells or a 10 μm width for cells with a diameter between 8 μm and 15 μm are optimized with respect to the target cell size and magnetization. The NiFe stripes are magnetized by the permanent magnet, leading to a high density field gradient around their edges, guiding the immunomagnetically labeled cells by balancing fluidic drag force and magnetophoretic force in order to focus the enriched cells^21^,^22^. In this way, the permanent magnet is not only used to fully magnetize the cells for detection but also to deterministically guide the cellular targets to the GMR stripes. Figure 1c shows a snapshot of optimally focused anti-CD4 labeled T-lymphocytes (false colored) upstream to the Wheatstone half-bridge. This *in situ* two-step cell enrichment and focusing technique implemented in the device allows for high recovery at rather large channel dimensions to avoid clogging when performing measurements in undiluted whole blood. For the applied 700 × 150 μm^2^ microfluidic channel cross section and 15 × 10 μm^2^ exit cross section, where the cells are expected to leave the NiFe chevrons for the sensing elements, we obtain an enrichment factor of 700x (Supplementary Movie 2 and 3).

By controlling magnetic and laminar drag forces, we are able to derive a number of cell characteristics from the magnetic signal pattern. First, we define the magnetic diameter as a signal attribute that can be directly correlated to the hydrodynamic diameter of the detected object (Fig. 1d). The magnetic diameter is defined as the distance between the minimum and maximum signal peak originating from one single GMR resistor. Data derived from numerical simulation shows the linear dependence between the magnetic diameter and the hydrodynamic diameter allowing for direct volumetric analysis. We define the dynamic range of the magnetic flow cytometer for a width of the GMR resistors of 2 μm as the span from 3 μm to 15 μm in hydrodynamic diameter where linear transformation of the magnetic diameter into the hydrodynamic diameter can be performed. The size resolution in this linear range is determined by the sampling rate of the read-out instruments and the velocity of the analyte passing the sensor. With a sampling rate of 10,000 samples s^−1^ and a cell velocity of 500 μm s^−1^ a spatial resolution of ~100 nm should be achievable. For the non-linear range below 3 μm and a sufficiently high sampling rate still TOF information can be acquired and we estimate the lower limit of size detection to be smaller than 1 μm. Second, the normalized integral of the signal pattern is directly related to cell size and can be calculated by summing up the absolute values of the integrals of the four signal half waves, each normalized by its peak value. Third, as the inset in Fig. 1d shows, the linear dependence between the measured signal amplitude and the magnetic moment readily allows the calculation of the number of bound MNPs on a single cell level. In detail, the magnetic coverage of the analyte is derived by incorporating sensor sensitivity, amplifier characteristics, and supply voltage, as well as nanoparticle magnetization into the analysis of the acquired magnetic signal from the rolling target cell.

### Validation of volumetric measurements

**Figure 2:**
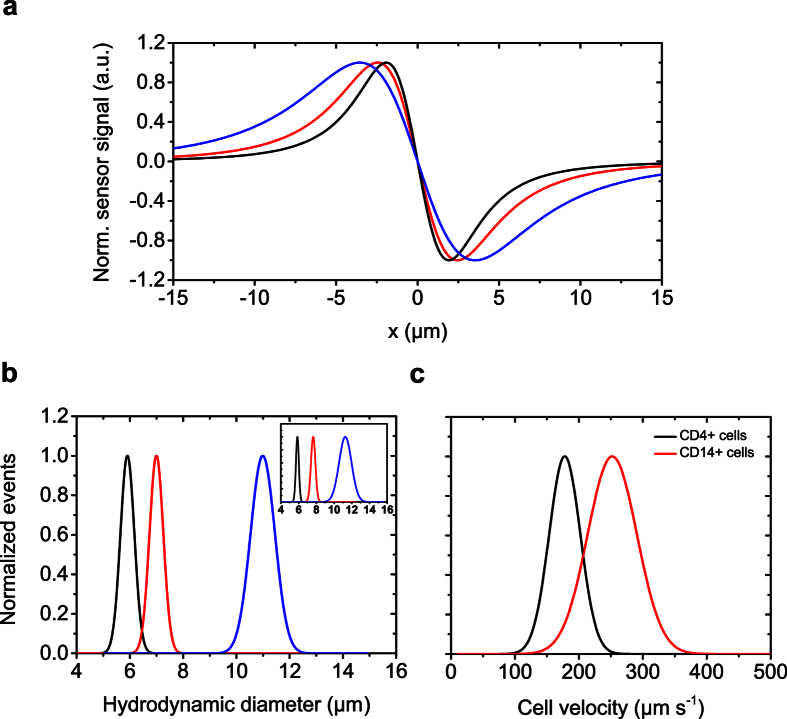
Proof of concept of volumetric measurements by magnetic TOF cytometry. (**a**) Numerically simulated dipole signals for 6 μm (black), 8 μm (red), and 12 μm (blue) spheres passing a single sensor stripe. (**b**) The same color code and magnetic bead sizes were used for volumetric discrimination and a benchmark against impedance measurements using a standard Beckman Coulter instrument (inset). The hydrodynamic diameter was derived from the normalized integral. (**c**) TOF differentiation of CD4 and CD14 immunomagnetically labeled leukocytes based on the velocity of the cell when passing the sensor.

For the analysis of the described cell characteristics by our TOF approach, we first explored volumetric measurements, since the calculation of derived parameters such as the number of MNPs bound to the analyte is based on the volumetric information. We first carried out numerical simulations of the magnetic stray fields for different bead sizes. Figure 2a depicts the normalized signals originating from a 6 μm, 8 μm, or 12 μm sphere with a magnetized core for a single GMR resistor. We observed that the magnetic diameter and the normalized integral both deliver distinct values for each hydrodynamic diameter. For experimental verification, samples of the monodispersed beads with a magnetic shell were prepared and measured at identical conditions with a flow rate of 1 μl s^−1^ that guaranteed precise *in situ* focusing for the given diameter range. After digitally low-pass filtering the signal with a cut-off frequency at 300 Hz, which is based on the rolling time of the analytes over the sensor area, cell detection and analysis were performed with a custom-made routine, identifying the characteristic signal pattern described above. For statistical analysis, we collected a minimum number of 115 events for each size, and the hydrodynamic diameter was then calculated using the normalized integral of the signal pattern. Mean values and standard deviations of the populations were derived by fitting the frequency counts with a Gaussian distribution (R^2^ > 0.90) (Fig. 2b). To verify the accuracy of the magnetically measured hydrodynamic diameter, we benchmarked aliquots of all samples with a Coulter instrument (Z2, Beckman Coulter), the gold standard for volumetric measurements (inset Fig. 2b). Both approaches are in good agreement for the mean values and standard deviations of the monodispersed particles. This measurement accuracy is obtained with a highly controlled rolling and positioning of the target analyte over the center of the GMR resistors. To demonstrate the analytical versatility of our instrument regarding clinical aspects, we carried out TOF measurements of immunomagnetically labeled T-lymphocytes and monocytes. Sample preparation was kept to a minimum by labeling the target cells directly in whole blood as described in the Methods section. When using functionalized MNPs to target the CD4 antigen, which is highly expressed on T-lymphocytes, the label is likely to address both T-lymphocytes and monocytes contained in the sample^23^. Most interestingly, no signal background from the monocytes was detected. This is related to the lower epitope density of CD4 on monocytes as compared to T-cells and measurement conditions with respect to flow rate to specifically detect T-lymphocytes. For this experiment we selected CD4 on the T-lymphocytes and CD14 on the monocytes as the target antigen^24^,^25^. Here, discrimination of the cell types can be based solely on the fluidic drag force acting on the immunomagnetically labeled cells. The monocytes experience a higher laminar drag force due to their larger hydrodynamic diameter and thus, show a mean velocity of 250 μm s^−1^ compared to the 177 μm s^−1^ for CD4^+^ lymphocytes. The mean values were derived from fitting the frequency values by a Gaussian distribution (R^2^ > 0.93) as illustrated in Fig. 2c.

### Considerations on sensor layout, vertical focusing, and magnetic labeling load

**Figure 3:**
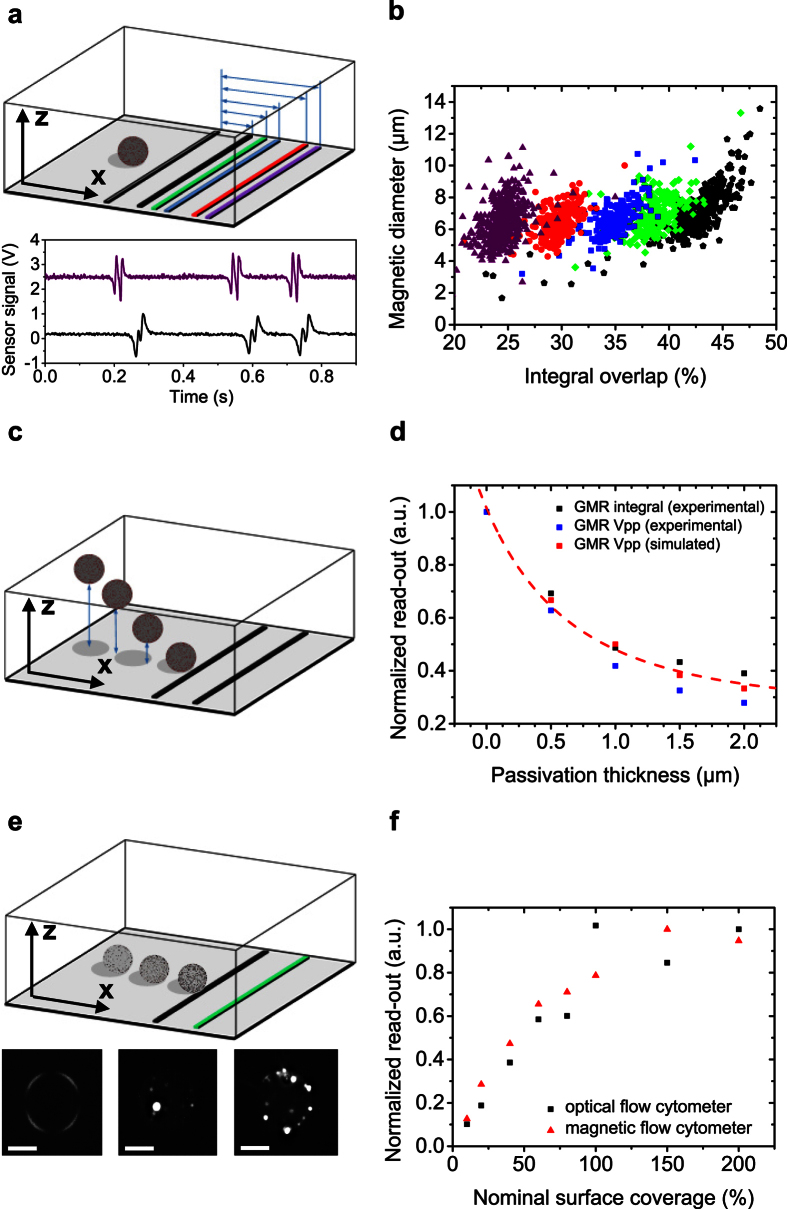
Analysis of the magnetic TOF concept. (**a**) Signal modulation as a function of hydrodynamic diameter and Wheatstone half-bridge layout. Signals are shown for a 12 μm bead magnetized on its surface passing a 22 μm (purple) and 10 μm (black) distance of the GMR sensors (same color code as in the schematics). With an increasing ratio of analyte to GMR resistor distance the inner peaks gradually overlap. (**b**) This overlap is quantified by the ratio of the outer to the inner peak integrals. By application of correction factors derived from numerical simulation, the magnetic diameter is extracted independently of the signal overlap. (**c**) Schematic illustration of the varied vertical offset of the analyte, which is experimentally induced by different passivation thicknesses in order to investigate the dependence of the magnetic signal on the vertical distance between analyte and sensor. (**d**) Correlation of experimentally determined sensor integral and peak-to-peak voltage for a 4 μm analyte with increasing offset of the analyte to 2 μm and a 6 μm sensor layout to the values derived from simulation under the same conditions. (**e,f**) To distinguish between different magnetic moments, we coupled different amounts of simultaneous superparamagnetic and fluorescent nanoparticles to polystyrene beads. Fluorescence images are shown for 0.02%, 38%, and 95% nominal surface coverage with MNPs (from left to right). In a titration experiment the magnetic flow cytometer was benchmarked with the fluorescence flow cytometer using the acquired peak-to-peak voltage of the magnetic sensor and the mean fluorescence intensity respectively. Scale bars are 5 μm.

For a deeper understanding of the measurement accuracy of the TOF methodology, we analyzed the limitations and opportunities for a precise calculation of the hydrodynamic diameter from the characteristic signal pattern. First, we analyzed the signal modulation introduced by an increasing ratio of the size of the analyte to the sensor layout. Figure 3a exemplarily shows distances of the half-bridge resistors and experimental results of 12 μm polymer beads with a magnetic shell acquired for a sensor configuration larger (22 μm, purple), and smaller (10 μm, black) than the hydrodynamic diameter of the analyte. With a shorter distance between the sensing elements, signal modulation is caused by simultaneous detection of the dipole field by the two resistors lowering the inner signal amplitudes (Supplementary Fig. 2a,b). Consequently, the positions of the inner peaks on the x-axis are shifted towards each resistor. To account for this, we introduce a correction factor for smaller sensor layouts derived from numerical simulation (Supplementary Fig. 3). Figure 3b shows the corrected data of the magnetic diameter for a 22 μm, 18 μm, 14 μm, 12 μm, and 10 μm sensor distance (from left to right). Furthermore, we take advantage of the signal overlap as a discriminator for a predetermined cell size. With a cell size to half-bridge ratio >1.5, we can even derive binary information with the proper selection of the half-bridge relative to the cell size threshold (Supplementary Fig. 2c–e).

A vertical z-offset between the sensor configuration and analyte, which could be due to hydrodynamic lift forces^26^,^27^, should be quantified in the following section. An increasing distance of the analyte to the sensing layer was artificially induced by variation of the thickness of the SiN passivation layer from 0.07 μm, as the standard layer thickness, to 2 μm, representing the maximum distance of the rolling cell to the GMR in our experiment (Fig. 3c). To investigate the concurrent reduction in the signal-to-noise ratio, we chose 4 μm analytes to maximize the ratio of analyte diameter to passivation thickness. For analysis of the signal intensity of the detected objects, we excluded GMR sensitivity variations by standardizing the calculated peak-to-peak voltages *GMR Vpp* and the sensor integral *GMR integral* by the transfer characteristics of the respective sensor. Additionally, numerical simulations with the same parameters were carried out and plotted with the experimental results in Fig. 3d. The decline of both signal voltage and integral is fitted with a 1/r^3^-dependence and is in good agreement with the simulated values for a theoretical dipole located at the center of the analyte. The results emphasize the importance of the rolling analytes and an ultra-thin passivation layer over the sensing elements for accurate volumetric measurements.

To examine the capability of our flow cytometer to measure magnetization load and therefore, nanoparticle binding capacity, we benchmarked our magnetic system against an optical flow cytometer (FACS Canto II, Becton Dickinson). Simultaneous magnetic and fluorescent MNPs, as described in the Methods section, allowed for analyzing the same sample with both instruments. To achieve different surface coverages, we calculated the respective amount of the MNPs by dividing the surface area of the polymeric bead by the area a single MNP occupies. We then added the calculated MNP volumes to a constant number of polymeric beads and incubated the sample to equilibrium state. Figure 3e shows labeled beads with 0.02%, 38%, and 95% theoretical surface coverage recorded with a fluorescence microscope at 500x magnification. Superposition of the binding curves for the mean fluorescence intensity and peak-to-peak voltages acquired with the optical flow cytometer and the magnetic system, respectively, shows very good agreement of both systems (Fig. 3f).

### Quantitative magnetic analysis on magnetically labeled erythrocytes

**Figure 4:**
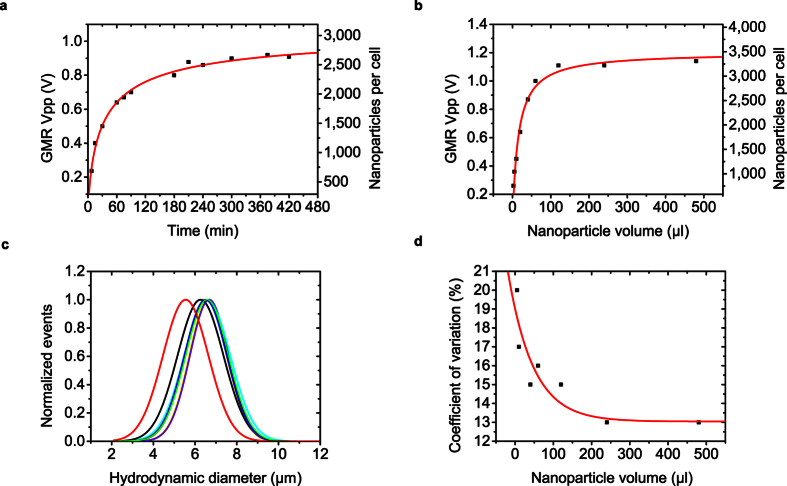
Binding experiments for erythrocytes as a biological model system for the magnetic flow cytometer. Titration curves are acquired for magnetic nanoparticles functionalized with anti-CD235a bound to erythrocytes for an increasing amount of (**a**) incubation time at a constant volume of labels and (**b**) labels at equilibrium. The peak-to-peak voltage of the sensor signal and calculated number of labels per cell is correlated to a sigmoidal Hill Fit. (**c**) Normalized distributions of the extracted hydrodynamic diameter for different amounts of magnetic moment attached to the cells. (**d**) The coefficient of variation as a means of the dispersion of the hydrodynamic diameter derived from the sensor output dependent on the amount of nanoparticles incubated with the sample.

Additional binding experiments were performed with red blood cells as model system. We prepared fresh blood collected by a venous puncture procedure for the preparation of spheroid erythrocytes. In addition, Coulter measurements were performed to verify cell concentration and cell volume. We determined the equilibrium time of the binding reaction by incubating 20 μl of diluted whole blood with 50 μl of the supplied stock solution containing the anti-CD235a functionalized MNPs (Fig. 4a). Prior to the calculation of the nanoparticle binding capacity, we validated our approach of calculating the magnetic moment attached to a single cell with a vibrating sample magnetometer, the Gold standard for magnetization measurements (Supplementary Fig. 4). With the magnetic moment of an individual MNP, as well as device parameters, we transferred the measured peak-to-peak voltage into the number of MNPs attached to an individual erythrocyte shown on the right y-axis in Fig. 4a. 90% of the signal intensity at saturation level was observed after 180 min, which was set as equilibrium time and used for the following kinetic experiments. We then varied the magnetic load attached to the erythrocytes by adding different volumes of MNP stock solution to the sample. From Fig. 4b, an amount of 100 μl stock solution of the beads is necessary to achieve MNP saturation to the erythrocytes. Subsequently, the nominal surface coverage of the erythrocytes can be calculated. Using a magnetic moment per MNP of 1.2 × 10^−17^ A m^2^ we derived a number of 3300 MNPs attached to an individual cell at saturation conditions. Using an MNP diameter of 75 nm as specified by the manufacturer and a mean hydrodynamic cell diameter of 6 μm derived from the sensor signal, a maximum nominal surface coverage of 14.4% is accomplished with a commercial cell separation kit. The acquired binding curves correlate well with a sigmoidal Hill fit (R^2^ > 0.98). Next, we investigated the calculation of the hydrodynamic diameter of the spheroid erythrocytes in dependence of the number of magnetic labels attached to a cell. Figure 4c shows the superposition of the hydrodynamic diameters for varying magnetic loads obtained by different volumes of MNP solution incubated with the sample at equilibrium conditions. We observe that the extraction of the hydrodynamic diameter based on a theoretical dipole model is independent of the magnetic load attached to the surface of the analyte. A significant deviation of the mean diameter from the majority of the populations is observed for a number of MNPs below ~1,150 bound per cell (black and red Gaussian fit). This can be attributed to an inaccurate approximation of the magnetic stray field outside the spheroid erythrocytes by the theoretical dipole model because the field lines for a low number of MNPs attached to the analyte deviate further from the dipole calibration model described by equation (3). The mean values and standard deviations were derived from a Gaussian fit applied to the raw data for each magnetic load. Figure 4d quantitatively shows the coefficient of variation as a measure of the mono-dispersity in dependence of the magnetic load by the coefficient of variation. It is observed that the coefficient of variation is in good agreement with characteristics of the acquired binding curve in Fig. 4b, and thus, measurement precision increases with magnetic load and homogenous label coverage. Furthermore, the magnetic flow cytometer potentially enables monitoring of labeling homogeneity (Supplementary Fig. 5).

### Discrimination of RBC morphology

**Figure 5:**
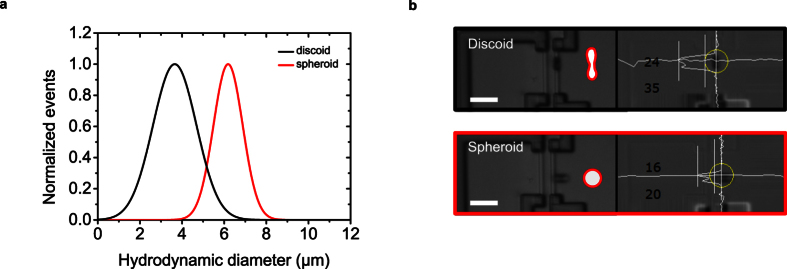
Red blood cell morphology analysis by magnetic signal pattern analysis. (**a**) Scan of the in-plane component of the magnetic field lines generated by an analyte passing the sensor area shows the feasibility of the magnetic flow cytometer to differentiate between magnetically labeled discoid and isovolumetrically sphered erythrocytes. The hydrodynamic diameter was derived from the magnetic diameter. (**b**) Time correlated microscope images and image analysis showing the different cell shapes passing the sensor with their characteristic orientation. Scale bars are 20 μm.

To investigate the capability of our flow cytometer to determine cell morphology information based on the magnetic fingerprint of an individual target cell, we prepared discoid and spheroid erythrocytes as described in the Methods section. Prior to incubation with 50 μl of MNPs directed against CD235a, the cell shape was controlled with an optical microscope at 500x magnification. Both samples were measured with an 8 μm sensor distance at an external magnetic field of 150 mT. Optical control of the cell passing the sensor was performed by time-correlated measurement of the magnetic signal and a microscope image of the sensing area (110 × 60 μm^2^) acquired at 30 frames per second. The analysis of the hydrodynamic diameter of the analytes of both morphologies was carried out using the magnetic diameter (Fig. 5a). The hydrodynamic diameter of 6.1 μm for the spheroid cells is in good correlation with the values obtained from impedance measurements (5.8 μm). However, as they are passing the sensing elements, the analysis of the discoid cells reveals a lower hydrodynamic diameter, which is assumed to be induced by their orientation as well as their field lines deviating from the dipole model used for calibration of our methodology (Fig. 5b).

## Discussion

We have developed a quantitative magnetic methodology utilizing cell rolling over a magnetic sensor to derive various cellular features based on the analysis of the characteristic magnetic fingerprint of an immunomagnetically labeled cell. In contrast to previous flow cytometry approaches utilizing a magnetic sensor array^13^,^14^, we achieve highly deterministic cell focusing by balancing fluidic and magnetophoretic forces which allows our magnetic flow cytometer to operate with a single Wheatstone bridge. In this way, we probe the surface of rolling cells on the sensor substrate, which mimics white blood cell rolling, e.g. along the endothelial cell layer of vessels in peripheral blood^28^. Most importantly, the precise positioning of the analyte relative to the sensing elements enables precise quantification of the hydrodynamic cell diameter. In addition, minimum passivation thickness between sensor and analyte increases the signal-to-noise ratio and minimizes the detection of coincidences (Supplementary Fig. 6).

We also benchmarked our system with respect to quantification of the magnetic labeling load bound to a single analyte against a vibrating sample magnetometer and a fluorescence flow cytometer. Contrary to earlier research on quantification of the binding capacity of immunomagnetically labeled cells^29^,^30^, our technique is not based on optical data acquisition and therefore, can even operate in opaque blood. The possibility of performing isovolumetric shape alteration on erythrocytes allowed us to even examine the capability of our magnetic flow cytometer to derive morphological information from the magnetic pattern of a single cell.

In brief, our measurement with negligible magnetic background does not rely on sample dilution, hemolysis, or other sample preparation, and purification steps and thus, can be applied to explore quantitative cell function testing based on hydrodynamic diameter, labeling homogeneity and density, morphology in close to *in-vivo* conditions. We even predict the potential of our flow cytometer to investigate cell-surface interactions by utilization of functionalized sensor substrates. Today, such a wealth of information cannot only be obtained with fluorescence flow cytometry but requires additional measurement techniques, such as impedance measurements, microscopy, and laminar flow chamber assays^28^,^31^,^32^. Our magnetic cell function analysis methodology could thus be highly attractive for clinical workflow integration and the valuable TOF information allows studying single cell interactions in opaque environments. This could be of interest to a broad range of clinical and research topics, which require a whole blood environment for accurate testing, such as immune competence testing or affinities of therapeutic antibodies. Last, our methodology could be of interest to quantify binding properties of immunomagnetic labels. Our future work will concentrate on the integration of the device with minimized Si footprint into a microfluidic cartridge incorporating sample incubation, cell focusing, quantitative analysis, and cell sorting. In addition, this miniaturized platform will potentially allow absolute cell counting, which is currently limited due to sedimentation effects in the tubing of the rapid prototype. The envisioned workflow potentially enables rapid and quantitative point-of-care testing of cell function in complex matrices at the bedside.

## Methods

### Assembly of the magnetic flow cytometer

The Si chips containing an array of 10 single sensors are manufactured by Sensitec GmbH (Lahnau, Germany). The singulated sensor chips are attached to a printed circuit board and wire-bonded for electrical connection to the peripheral components. For sample transport over the sensing elements, a microfluidic channel made out of polydimethylsiloxane (PDMS) is positioned over the sensor array. Standard soft photolithography with an epoxy resin (SU-8, 2025, MicroChem Corp.) is utilized to fabricate a negative master mold on a Si substrate as described elsewhere^33^. Ports for inlet and outlet are punched into the PDMS microfluidics before device assembly. The fluidics is then inked for 5 seconds on a thin layer of uncured PDMS spun onto a Si substrate before aligning the channel onto the sensor chip using a stereo microscope. Last, the assembled device is cured at 60 °C for 120 min resulting in a permanent bond between the sensor chip and microfluidics. Stable laminar flow conditions are established by a pulsation-free syringe system (Nemesys, cetoni GmbH) connected *via* a PVC tubing (inner diameter 0.5 mm, ZEFA-Laborservice GmbH) to the microfluidics (Supplementary Figure 7).

### Detection system setup

The sensor is supplied by an AC modulated signal (HP 33120A, Hewlett-Packard GmbH) with a frequency of 13 kHz and a peak-to-peak voltage of up to 1.1 V. For the electrical readout the differential signal of the Wheatstone bridge is fed into a lock-in setup (LIA-MVD-200-H, Femto Messtechnik GmbH) that amplifies its input 30,000-fold while averaging the output signal with a time constant of 300 μs. The processed signal is then digitized by a LabVIEW data acquisition board (PCI 6251, National Instruments Corp.) at a sample rate of 10 kS s^−1^ and 16 Bit. Optical control and correlation of the magnetic sensing is performed with a customized Leica microscope with 200x magnification and a CCD camera system (TXG 14F, Baumer GmbH) which is implemented in the LabVIEW control panel with an acquisition rate of 30 frames per second. The non-magnetic peripheral components of the setup are arranged around the NdFeB permanent magnet generating a vertical magnetic field density B_external_ within a range between 100 mT and 170 mT measured at the magnet center with a Gauss meter (CYHT201, ChenYang Technologies GmbH).

### Signal identification and analysis

Detection of the characteristic signal pattern in a continuous measurement is performed with a home-made algorithm implemented into a state-event machine. First, the sensor signal is passed through a Finite Impulse Response low-pass filter with a cut-off frequency depending on the time span of the peak pattern from a single analyte. Second, the amplitudes and data points between two adjacent peaks are calculated and fed into the state-event machine which determines if neighboring peaks, whose peak-to-peak amplitude is exceeding a given threshold, originate from an analyte passing the sensor area. Therefore the algorithm probes the continuous measurement with a predefined time frame, based on the signal duration, for characteristic four peak patterns. To derive the sensor integral, the beginning and ending of the signal are defined at the time the signal drops to 25% of its maximum peak value. The absolute of the integral is then calculated for each half wave and the summed up for the *sensor integral*. The *normalized integral* is determined by summing up the absolute of the integral of the half waves, each normalized to its maximum peak value. Based on calibration data for the magnetic diameter as well as the normalized integral derived from numerical simulation of the sensor signal, as shown in Fig. 1d, the hydrodynamic diameter is calculated from the magnetic fingerprint of the analyte. The hydrodynamic diameter can either be derived from the magnetic diameter using a linear fit or from the normalized integral using a 3^rd^ order polynomial fit of the calibration data (Supplementary Figure 8). The term *normalized sensor signal* refers to the *sensor signal* normalized to its maximum value for visualization. The term *sensor signal* represents the original voltage output of the Wheatstone half-bridge derived from numerical simulation and the experimental setup, respectively. The number of bound magnetic nanoparticles is calculated using the radius of the analyte derived from the Time-of-flight data, which corresponds to the center position of the analyte *r*_*dipole*_ in equation (3) describing the magnetic dipole field ***B***_*dipole*_ used for calibration. The magnetic moment *m*_*dipole*_ of the analyte is then derived from numerical simulation data for the given *r*_*dipole*_. Last, *m*_*dipole*_ is divided by the individual magnetic moment of a single MNP in order to obtain the number of MNPs bound to a single analyte.

### Reference microspheres for validation of the system

For reference experiments we used polystyrene beads coated with a superparamagnetic iron oxide shell (micromer^®^-M, micromod Partikeltechnologie GmbH). The beads show a magnetization and size comparable to that expected from an immunomagnetically labeled cell and are utilized to validate the magnetic flow cytometry approach with respect to cell enrichment, focusing, and signal analysis. As a model system we chose simultaneous magnetic and fluorescent biotinylated MNPs with a diameter of 200 nm (nano-screenMAG/G-Biotin, chemicell GmbH) and 12 μm polystyrene beads functionalized with streptavidin due to its rapid and strong bond formation.

### Biological assays and functionalized magnetic nanoparticles

For whole blood assay experiments we collected fresh EDTA blood from healthy donors to demonstrate measurements of CD4^+^ T-lymphocytes and CD14^+^ monocytes using a commercial labeling kit consisting of MNPs functionalized with specific antibodies directed against the respective cell type (MACS whole blood MicroBeads, Miltenyi Biotec GmbH). To determine the value of the magnetic moment of a single MNP we analyzed 40 μl aliquots of the MNP suspension with a vibrating sample magnetometer. With the known concentration of MNPs in the stock solution we calculated a saturation magnetic moment of 1.2 10^−17^ Am^2^ per MNP. The only sample preparation step for magnetic flow cytometry analysis consisted of the incubation of 100 μl of whole blood with 50 μl stock solution of the magnetic nanoparticles for 180 min at room temperature. Subsequently, the opaque sample was diluted with Dulbecco’s phosphate buffered saline (PBS, life technologies GmbH) at a ratio of 1:50 to perform time-correlation of optical and magnetic measurement. As a biological reference assay we selected erythrocytes due to their small volume variation and the controlled cell shape alteration^34^,^35^. Isovolumetrically sphered cells were obtained by preparing a 1:600 dilution with sphering buffer (ADVIA R120 RBC/PLT, Siemens Healthcare GmbH). Discoid erythrocytes were prepared by diluting whole blood by a factor of 1:600 with 7.39 mmol EDTA, 109.3 mmol NaCl and 0.11% glutaraldehyde dissolved in PBS. For magnetic targeting anti-CD235a functionalized MNPs (MACS MicroBeads, Miltenyi Biotec GmbH) were used.

## Additional Information

**How to cite this article**: Reisbeck, M. *et al*. Magnetic fingerprints of rolling cells for quantitative flow cytometry in whole blood. *Sci. Rep.* **6**, 32838; doi: 10.1038/srep32838 (2016).

## Supplementary Material

Supplementary Movie 1

Supplementary Movie 2

Supplementary Movie 3

Supplementary Information

## References

[b1] Robinson J. P. & Roederer M. Flow cytometry strikes gold. Science 350, 739–740 (2015). 26564833 10.1126/science.aad6770

[b2] Bendall S. C. . Single-cell mass cytometry of differential immune and drug responses across a human hematopoietic continuum. Science 332, 687–696 (2011). 21551058 10.1126/science.1198704 PMC3273988

[b3] Göhde W. . Individual patient-dependent influence of erythrocyte lysing procedures on flow-cytometric analysis of leukocyte subpopulations. Transfus, Med. Hemother. 30, 165–170 (2003).

[b4] Greve B. . High-grade loss of leukocytes and hematopoietic progenitor cells caused by erythrocyte lysing procedures for flow cytometric analyses. J Hematother Stem Cell Res. 12, 321–330 (2003). 12857373 10.1089/152581603322023052

[b5] Baselt D. R. . A biosensor based on magnetoresistance technology. Biosens. Bioelectron. 13, 731–739 (1998). 9828367 10.1016/s0956-5663(98)00037-2

[b6] Osterfeld S. J. . Multiplex protein assays based on real-time magnetic nanotag sensing. Proc. Nat. Acad. Sci. USA 105, 20637–20640 (2008). 19074273 10.1073/pnas.0810822105 PMC2602607

[b7] Bechstein D. . High performance wash-free magnetic bioassays through microfluidically enhanced particle specificity. Sci. Rep. 5, 11693 (2015). 26123868 10.1038/srep11693 PMC4485157

[b8] Henriksen A. D., Wang S. X. & Hansen M. F. On the importance of sensor height variation for detection of magnetic labels by magnetoresistive sensors. Sci. Rep. 5, 12282 (2015). 26195089 10.1038/srep12282 PMC4508666

[b9] Wang W. . Magnetoresistive performance and comparison of supermagnetic nanoparticles on giant magnetoresistive sensor-based detection system. Sci. Rep. 4, 5716 (2014). 25043673 10.1038/srep05716 PMC4104391

[b10] Gaster R. S. . Quantification of protein interactions and solution transport using high-density GMR sensor arrays. Nature Nanotechnol . 6, 314–320 (2011). 10.1038/nnano.2011.45 PMC3089684 21478869

[b11] Schotter J., Shoshi A. & Brueckl H. Development of a magnetic lab-on-a-chip for point-of-care sepsis diagnosis. J. of Mag. and Mag. Mat. 321, 1671–1675 (2009).

[b12] Helou M. J. . Time-of-flight magnetic flow cytometry in whole blood with integrated sample preparation. Lab Chip 13, 1035–1038 (2013). 23392232 10.1039/c3lc41310a

[b13] Loureiro J. . Magnetoresistive chip cytometer, Lab Chip 11, 2255–2261 (2011). 21562656 10.1039/c0lc00324g

[b14] Issadore D. . Ultrasensitive clinical enumeration of rare cells ex vivo using a micro-hall detector. Sci. Transl. Med. 4, 141ra92 (2012). 10.1126/scitranslmed.3003747 PMC3603277 22764208

[b15] Lee C. P., Lai M. F., Huang H. T., Lin C. W. & Wei Z. H. Wheatstone bridge giant-magnetoresistance based cell counter. Biosens. Bioelectron. 57, 48–53 (2014). 24534580 10.1016/j.bios.2014.01.028

[b16] Lee H., Shin T. H., Cheon J. & Weissleder R. Recent developments in magnetic diagnostic systems. Chem. Rev. 115, 10690–10724 (2015). 26258867 10.1021/cr500698d PMC5791529

[b17] Issadore D. . Magnetic sensing technology for molecular analyses. Lab Chip 14, 2385–2397 (2014). 24887807 10.1039/c4lc00314d PMC4098149

[b18] Hayden, O. & Rührig, M. Inventors; Siemens AG, assignee; Device and method for concentrating and detecting magnetically marked cells in laminarly flowing media. European patent EP 2,404,155. 2012 Jan 11.

[b19] Jackson J. D. Classical Electrodynamics 3rd Edition, Wiley, Section 5 (1999).

[b20] Hayden O., Helou M. J., Reisbeck M. & Tedde S. F. Inventors; Siemens AG, assignee; Magnetic flow cytometry for high sample throughput. European patent EP 2,641,087. 2016 Jan 20.

[b21] Inglis D. W., Riehn R., Austin R. H. & Strum J. C. Continuous microfluidic immunomagnetic cell separation. Appl. Phys. Lett. 85, 5093–5095 (2004).

[b22] Xia N. . Combined microfluidic-micromagnetic separation of living cells in continuous flow. Biomed. Microdev. 8, 299–308 (2006). 10.1007/s10544-006-0033-0 17003962

[b23] Kazazi F., Mathjis J. M., Foley P. & Cunningham A. L. Variations in CD4 expression by human monocytes and macrophages and their relationship to infection with the human immunodeficiency virus. J. gen. Virol. 70, 2661–2672 (1989). 2677236 10.1099/0022-1317-70-10-2661

[b24] Davis K. A., Abrams B., Iyer S. B., Hoffman R. A. & Bishop J. E. Determination of CD4 antigen density on cells: role of antibody valency, avidity, clones, and conjugation. Cytometry 33, 197–205 (1998). 9773880 10.1002/(sici)1097-0320(19981001)33:2<197::aid-cyto14>3.0.co;2-p

[b25] Jersmann H. Time to abandon dogma: CD14 is expressed by non-myeloid lineage cells. Immunol. and Cell Bio. 83, 462–467 (2005). 16174094 10.1111/j.1440-1711.2005.01370.x

[b26] Hejazian M., Weihua L. & Nguyen N. T. Lab on a chip for continuous-flow magnetic cell separation. Lab Chip 15, 959–970 (2015). 25537573 10.1039/c4lc01422g

[b27] Patankar N. A., Huang P. Y., Ko T. & Joseph D. D. Lift-off of a single particle in Newtonian and viscoelastic fluids by direct numerical simulation. J. Fluid. Mech. 438, 67–100 (2001).

[b28] Sperandio M., Pickard J., Unnikrishnan S., Acton S. T. & Ley K. Analysis of leukocyte rolling *in vivo* and *in vitro*. Methods Enzymol. 416, 346–371 (2006). 17113878 10.1016/S0076-6879(06)16023-1

[b29] McCloskey K. E., Chalmers J. J. & Zborowski M. Magnetophoretic mobilities correlate to antibody binding capacities. Cytometry 40, 307–315 (2000). 10918281 10.1002/1097-0320(20000801)40:4<307::aid-cyto6>3.0.co;2-h

[b30] McCloskey K. E., Comella K., Chalmers J. J., Margel S. & Zborowski M. Mobility measurements of immunomagnetically labeled cells allow quantitation of secondary antibody binding capacity. Biotechnol. Bioeng. 75, 642–655 (2001). 11745142 10.1002/bit.10040

[b31] Faraasen S. . Ligand-Specific targeting of microspheres to phagocytes by surface modificytion with poly(l-lysine)-grafted poly(ethylene glycol) conjugate. Pharm. Res. 20, 237–246 (2003). 12636162 10.1023/a:1022366921298

[b32] Pierres A., Touchard D., Benoliel A.-M. & Bongrand P. Dissecting Streptavidin-Biotin Interaction with a laminar flow chamber. Biophys. J. 82, 3214–3223 (2002). 12023246 10.1016/S0006-3495(02)75664-6 PMC1302111

[b33] Xia Y. & Whitesides G. M. Soft Lithography. Angew. Chem. Int. Ed. 37, 550–575 (1998). 10.1002/(SICI)1521-3773(19980316)37:5<550::AID-ANIE550>3.0.CO;2-G 29711088

[b34] Kim Y. R. & Ornstein L. Isovolumetric sphering of erythrocytes for more accurate and precise cell volume measurement by flow cytometry. Cytometry 3, 419–427 (1983). 6851791 10.1002/cyto.990030606

[b35] Rudenko S. V. Erythrocyte morphological states, phases, transitions and trajectories. Biochim. Biophys. Acta. 1798, 1767–1778 (2010). 20538541 10.1016/j.bbamem.2010.05.010

