# Supplementary material for: Magnetic fingerprints of rolling cells for quantitative flow cytometry in whole blood
Source: Sci Rep. 2016 Sep 6;6:32838. doi: 10.1038/srep32838 (PMC5011763; doi:10.1038/srep32838)
Supplement: Supplementary Information [file srep32838-s4.pdf]

Supplementary Information for "Magnetic fingerprints of rolling cells for quantitative flow cytometry in whole blood"

Mathias Reisbeck*[1,2], Michael Johannes Helou[1], Lukas Richter[1], Barbara Kappes[2], Oliver Friedrich[2] & Oliver Hayden*[1]

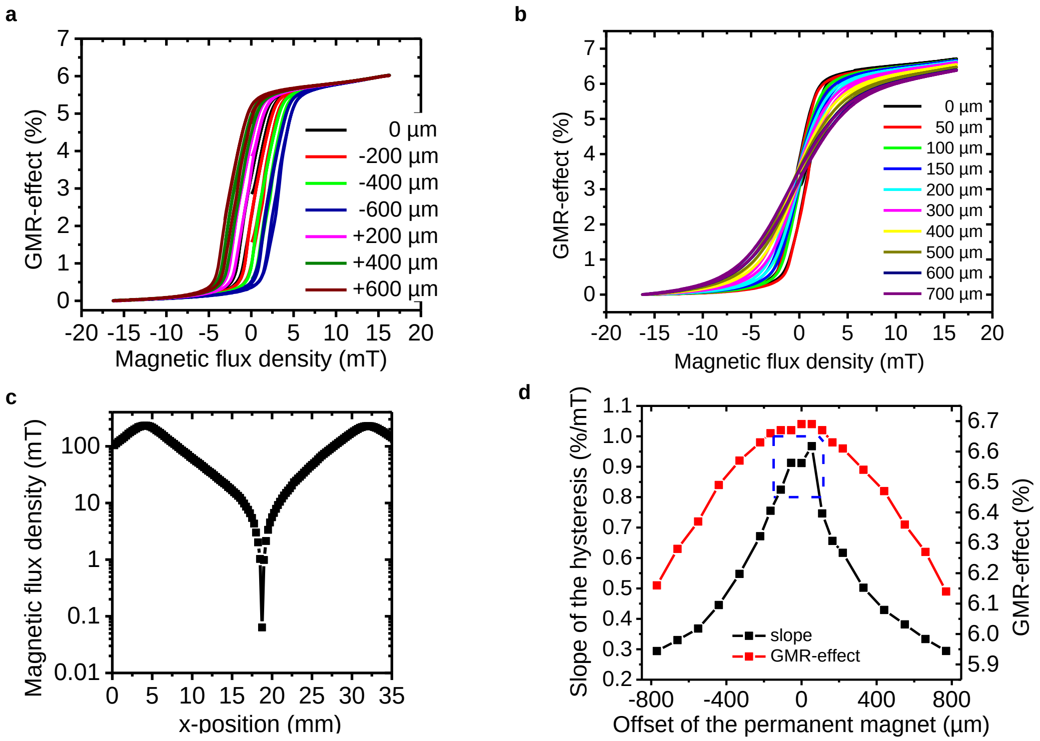

Supplementary Figure 1 | Precise positioning of the permanent magnet for cell detection. In order to achieve reproducible and precise volumetric analysis with a high signal-to-noise ratio, a well-defined and stable transfer curve of the sensor curve is needed. Thus, we utilize a Helmholtz coil setup generating an altering magnetic in-plane field component. While measuring the current flowing through the sensor configuration, we adjust the position of the permanent magnet in x- and y-dimension by a tunable optical stage. (**a**) By positioning the permanent magnet in the x-direction, we eliminate in-plane field components, which bias the sensing layer with an angle relative to the hard magnetic layer. (**b**) By minimizing y-components of the permanent magnet, we compensate for a pinning of the sensing layer along the resistor and therefore ensure maximal sensitivity. (**c**) Mapping of the magnetic field density of the permanent magnet in x-direction indicates the minimum position located at the center of the magnet. (**d**) Slope of the hysteresis curve in its linear regime at magnetic zero field and GMR effect of the sensing elements dependent on the offset in y-position. The blue box indicates the tolerance of the position in y-position, which is roughly +/- 150 µm, wherein 80 % of the initial slope and effect is obtained.

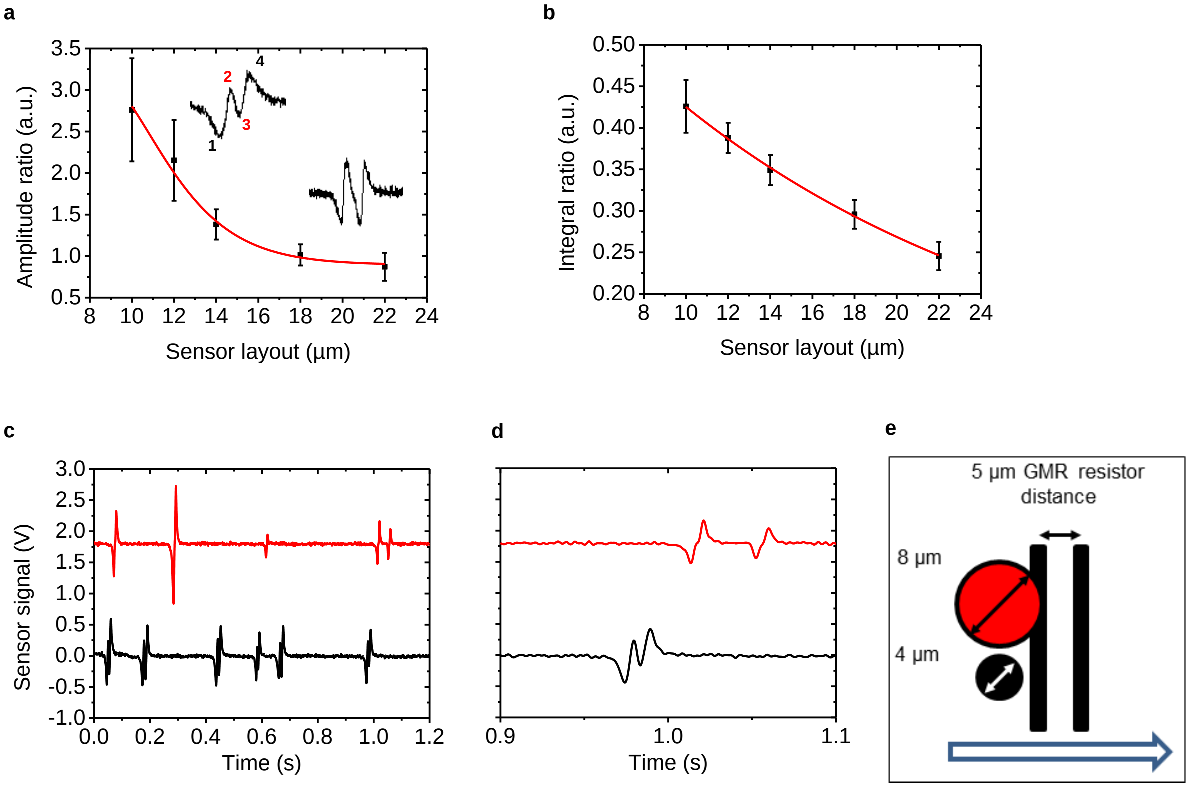

Supplementary Figure 2 | Analysis of the signal modulation and hardware based discrimination of the hydrodynamic diameter. Amplitude ratio of peak 1 and peak 2 (**a**) and ratio of the integral values of the first and second signal peak (**b**), dependent on the sensor layout, quantify the signal modulation induced by a decreasing sensor distance at constant hydrodynamic diameter of the analyte, which was chosen to be 12 µm in this experiment. (**c**) Measurements were performed with 4 µm and 8 µm reference beads with a magnetized shell on 5 µm sensor layout under identical conditions. (**d**) Detailed analysis of two 8 µm particles passing the sensor reveals that the inner signal peaks are vanished completely due to simultaneous detection of the dipole field of the analyte by both GMR resistors. However, the 4 µm analyte forms the characteristic four-peak-pattern. The discrimination of the diameter can therefore be based solely on the sensor layout automatically sorting analytes with a diameter above a threshold of ~1.5 (hydrodynamic diameter / sensor layout). (**e**) Schematic of the experiment true to scale.

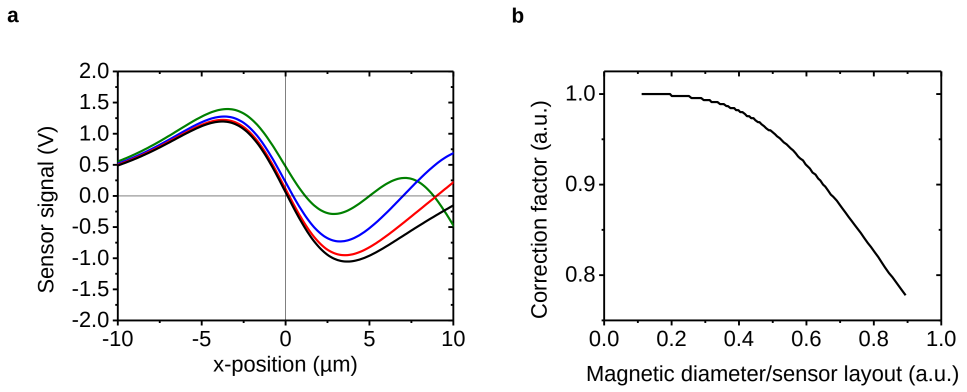

Supplementary Figure 3 | Deriving correction factors from numerical simulation. (**a**) Simulated signals of cell with a hydrodynamic diameter of 13 µm passing sensor layouts with a distance of 10 µm (green), 14 µm (blue), 18 µm (red), and 22 µm (black). The sensing element of the Wheatstone half-bridge generating the two peaks is located at a x-position of 0 µm. With increasing signal overlap, the position of the signal peaks is shifted towards the resistor. This results in a miscalculation of the velocity and the hydrodynamic diameter of the cell. (**b**) Therefore, the magnetic diameter derived from the distances between the positions of the peaks is corrected by a factor determined by numerical simulation. The factor is chosen dependent on the ratio of the initial magnetic diameter and the sensor layout.

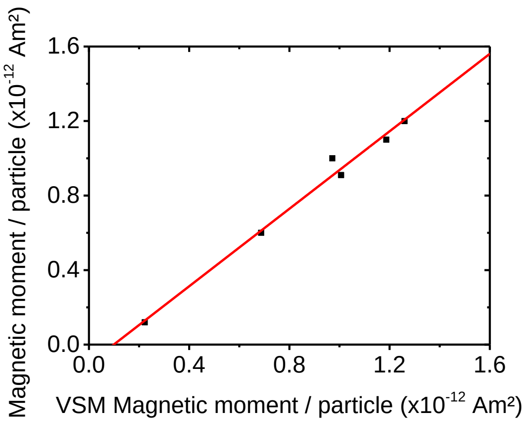

Supplementary Figure 4 | Calibration of the magnetic flow cytometer with customized reference beads. 8 µm particles with a defined magnetic content per weight (12 %, 35 %, 53 %, 73 %, 93 %, 100 %) were obtained from Micromod® Partikeltechnologie to benchmark the magnetic moment per analyte derived with the magnetic flow cytometer against a vibrating sample magnetometer (VSM, PMC MicroMag 3900 Series, Lakeshore Cryotronics). Therefore aliquots were prepared for each magnetic load and analyzed with a VSM. The magnetic moment is calculated on a single cell level with the magnetic flow cytometer whereas the VSM had to take the particle concentration specified by the manufacturer into account. The magnetic moments per analyte of the different samples obtained from both instruments are in good agreement.

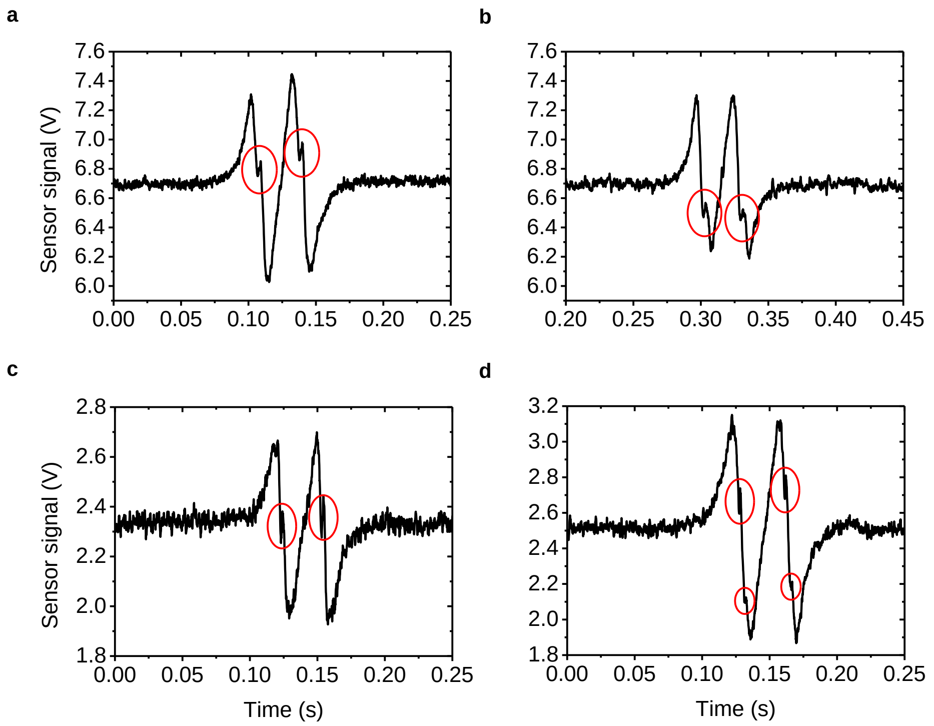

Supplementary Figure 5 | Evaluating labeling homogeneity with reference beads. Streptavidin coated 12 µm beads were labeled with a varying amount of biotinylated MNPs and measured with a 22 µm sensor layout. For a nominal surface coverage greater than 80 %, additional peaks can be observed between the first and second as well as between the third and fourth peak (**a**-**d**). As the peaks are almost symmetric for the signals originating from each resistor, it is assumed that the peaks are caused by MNP aggregates bound to the analyte. This assumption can be confirmed by the peaks being located between two peaks attributed to one resistor. This position is correlated to the analyte being positioned directly above the sensor, where there is a minimal distance between the MNPs and the sensor element. In this configuration, the stray field of the cell cannot be approximated by a dipole field due to the MNP aggregates as the dominant part of the magnetic ensemble.

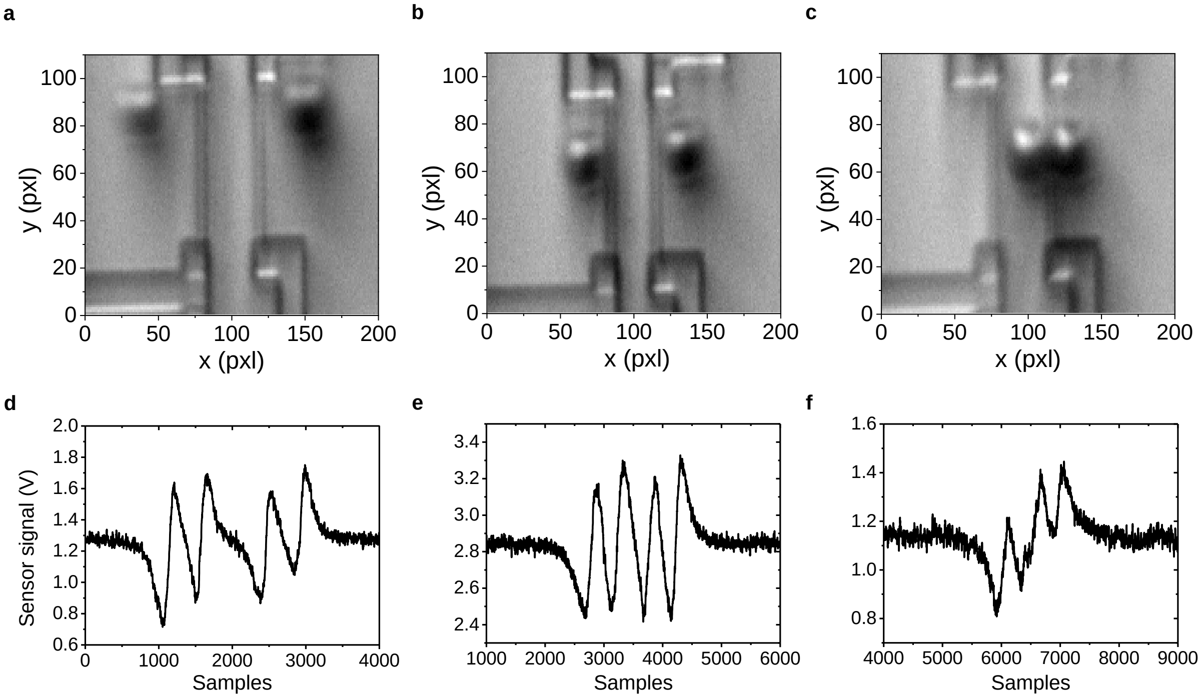

Supplementary Figure 6 | Doublette detection with the magnetic flow cytometer. Optical correlation of two 12 µm beads sequentially passing a sensor layout of 16 µm. A distance of ~55 µm between the center of the analytes ensures complete separation of the two four-peak-patterns (**a**&**d, b**&**e**). Two directly attached analytes generate a six-peak-pattern due to the interfering dipole field vectors (**c**&**f**). The minimum distance required for two completely separated four-peak-patterns over a 16 µm sensor layout is determined to be ~33 µm.

a b

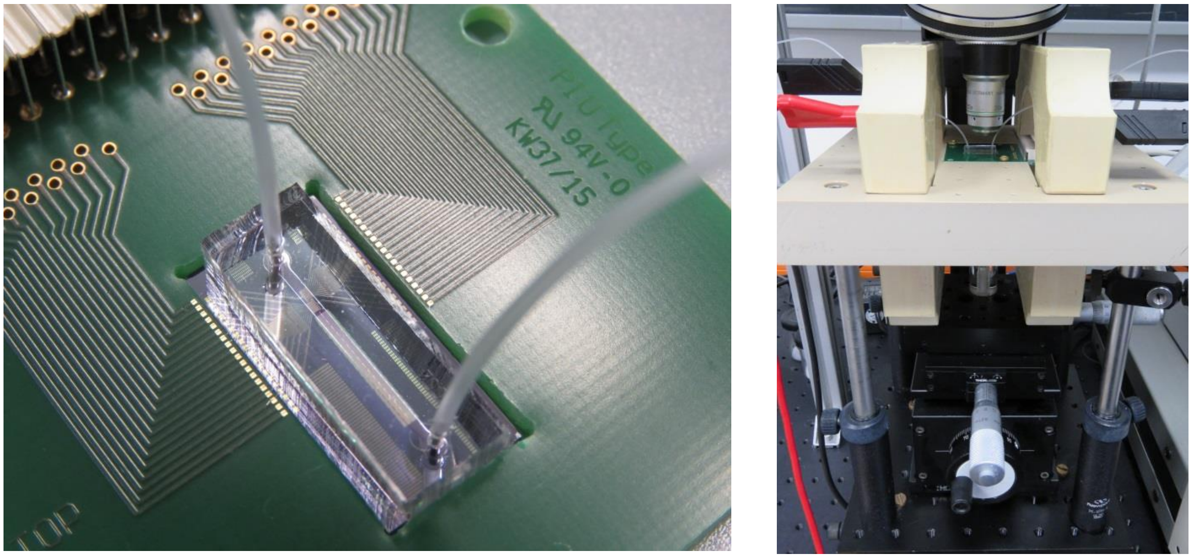

Supplementary Figure 7 | Lab prototype and setup. (**a**) 2x1 cm² sensor chip and microfluidics connected to a PVC tubing for sample transport over the integrated magnetophoretic patterns. The sensor chip is mounted on a PCB board and wire bonded for electrical connection. (**b**) The lab prototype is placed in a sensor holder and a NdFeB permanent magnet is positioned with a x/y/z stage. Additional Helmholtz coils are used to characterize the GMR hysteresis and the positioning of the magnet. For optical correlation of the magnetic response a demagnetized microscope objective is placed above the microfluidic for reflection microscopy.

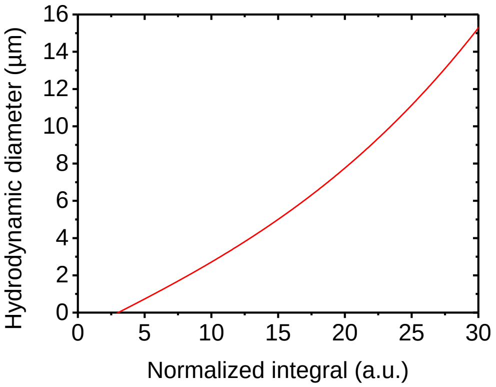

Supplementary Figure 8 | Calibration of the normalized integral. Numerical simulation is performed for a hydrodynamic diameter up to 16 µm over an 18 µm sensor layout and the normalized integral is derived from the signals. The data is fitted with a 3rd order polynomial fit, which allows for calculation of the hydrodynamic diameter from the sensor signal.

Supplementary Videos for "Magnetic fingerprints of rolling cells for quantitative flow cytometry in whole blood"

Mathias Reisbeck*[1,2], Michael Johannes Helou[1], Lukas Richter[1], Barbara Kappes[2], Oliver Friedrich[2] & Oliver Hayden*[1]

Supplementary Video 1: Cell rolling to probe the magnetic fingerprint

Supplementary Video 2: Precise cell focusing by magnetophoretic means

Supplementary Video 3: Enrichment and detection of monocytes labeled in whole blood
